# Bone Metastasis from Renal Cancer Coinciding with the Same Anatomical Position as a Vertebral Hemangioma: A Collision Lesion Case Report

**DOI:** 10.1055/s-0044-1779747

**Published:** 2024-03-28

**Authors:** André Marcondes Braga Ribeiro, Amanda Silles Borin, Guilherme Dias Rocha de Mello, Rubens Chojniak

**Affiliations:** 1 Department of Nuclear Medicine A.C.Camargo Cancer Center, São Paulo, SP, Brazil; 2 Department of Radiology, A.C.Camargo Cancer Center, São Paulo, SP, Brazil

**Keywords:** bone metastasis, collision lesion, FDG, PET/CT, renal cancer, vertebral hemangioma

## Abstract

Collisions lesions are rare neoplasms where two histologically distinct tumors coexist in the same organ or anatomical site. Vertebral hemangiomas (VHs) are the most common lesions involving the vertebral bodies and imaging findings of typical and atypical hemangiomas, variant forms of hemangioma such as aggressive hemangiomas are well known, but collision lesions involving VHs are extremely rare. This article presents a case report of a 73-year-old male patient diagnosed with clear cell renal cancer in a rare presentation of a bone metastasis coinciding with the same anatomical position as a VH (collision lesion). This required a multidisciplinary approach involving various diagnostic techniques to determine the best therapeutic management.

## Introduction

Renal cancer (RC) is the ninth most common cancer worldwide, with approximately 431,000 new cases annually, and the most frequent subtype is clear cell carcinoma (ccRC). The highest incidence rates are found in Eastern Europe and the United States. 1

Most patients present with localized disease amenable to surgery for definitive treatment, but 30% of patients may have metastatic disease at the initial presentation. Additionally, about one-third of patients treated with curative intent will develop metastatic disease. 2 The most common distant metastases are to the bones, lungs, liver, and brain. 3

The staging of RC is performed using the TNM system, considering the tumor size (T), regional lymph node metastasis (N), and distant metastasis (M). Imaging exams such as computed tomography (CT), magnetic resonance imaging (MRI), ultrasonography, and bone scintigraphy are used for this classification. 4

Bone metastases tend to appear in MRI as hypointense areas in T1-weighted images and hyperintense areas in T2-weighted images, with enhancement after contrast administration. 5 On the other hand, typical hemangiomas, due to their rich fatty stroma, appear hyperintense on T1 and T2 sequences with homogeneous signal suppression on fat-saturated sequences. Atypical hemangiomas, due to the increased proportion of vascular channels, interstitial edema, and relatively low-fat content, demonstrate atypical appearances on MRI. 6

Our case report revolves around a 73-year-old male patient diagnosed with ccRC presenting a rare manifestation—a bone metastasis coinciding with a vertebral hemangioma (VH; collision lesion). The intricate diagnostic journey involved various imaging modalities, including CT, MRI, and specialized scans such as 18F-labeled fluorodeoxyglucose positron emission tomography/computed tomography (FDG-PET) and technetium-99m-labeled red blood cells scintigraphy (RBCS). The patient also participated in a research protocol for 18F-labeled prostate-specific membrane antigen prostate-1007 positron emission tomography/computed tomography (PSMA-PET).

Collision lesions are rare neoplasms where two histologically distinct tumors coexist in the same organ or anatomical site and are often described in the hepatobiliary system, genitourinary system and adrenal glands. VHs are the most common lesions involving the vertebral bodies, with an incidence of 9 to 12% in adults. 7 VHs are usually asymptomatic and rarely they can be symptomatic; however, when they occur in the thoracic vertebrae, they are more likely to present symptoms due to the narrow dimensions of the vertebral canal, requiring more aggressive management before onset of serious neurological sequelae. Therefore, it is typically advised that these lesions are not surgically manipulated. 8 Imaging findings of typical and atypical hemangiomas, variant forms of hemangioma such as aggressive hemangiomas are well known, but collision lesions involving VHs are extremely rare. 7

The consideration of hypotheses such as atypical/aggressive VH or a “collision lesion” underscores the complexity of this diagnostic challenge. The following paragraphs delve into the specific imaging characteristics and findings that led to these considerations and the subsequent confirmation through biopsy, paving the way for targeted treatment.

This case report not only sheds light on the intricacies of diagnosing rare presentations like “collision lesions” but also emphasizes the indispensable role of a multidisciplinary approach in navigating such complex diagnostic landscapes in oncology.

## Case Presentation

**Fig. 1:**
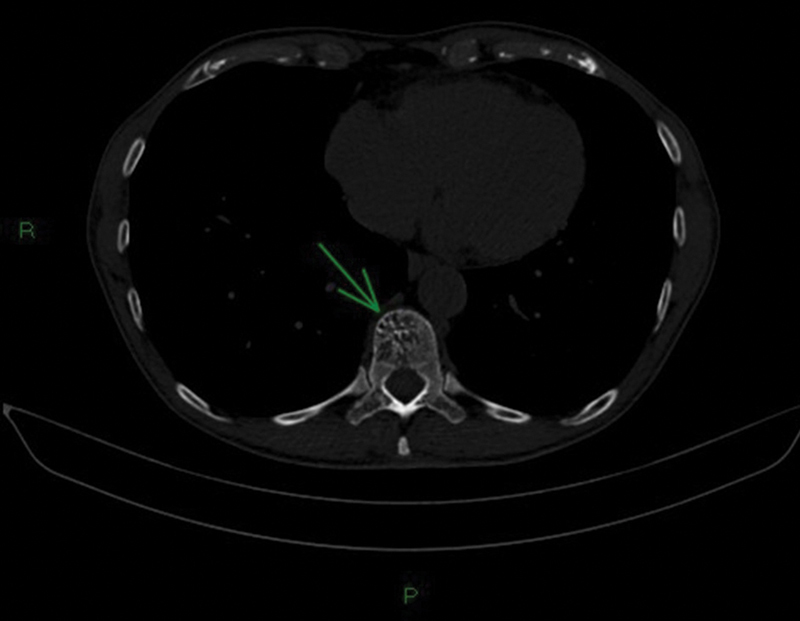
Axial slice image showing the so-called “polka-dot” or “salt and pepper” sign in which cross-section of the vertebra on computed tomography shows foci of high density owing to trabecular thickening, a finding characteristic of hemangiomas (green arrow).

**Fig. 2:**
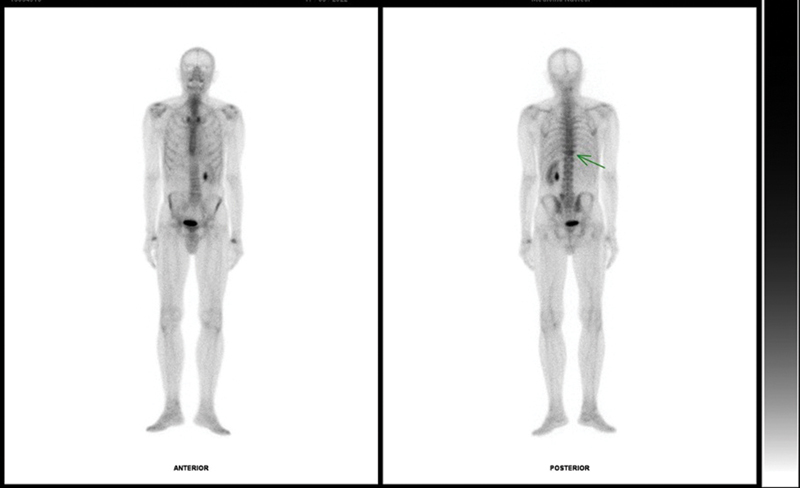
Bone scintigraphy for staging demonstrating an area of mild hyperconcentration of the radiotracer in the projection of the T11 vertebra ( *green arrow* ), consistent with the area observed in the computed tomography scan.

A 72-year-old male patient was diagnosed with a suspicious heterogeneous mass in the right kidney during routine exams in early 2022. Subsequent staging exams were performed. In the chest CT scan of March 2022, signs of hemangiomas in the thoracic vertebral bodies of T5 and T11 were evident ( Fig. 1 ). A bone scintigraphy at the same time detected areas of slight radiotracer uptake in the same thoracic vertebrae ( Fig. 2 ). Abdominal and pelvic MRI revealed the local lesion associated with renal vein and vena cava thrombosis, with no signs of lymphadenopathy.

In April 2022, the patient underwent right robotic radical nephrectomy with cavotomy, and the anatomopathological diagnosis demonstrated a ccRC, International Society of Urological Pathology (ISUP) grade 4., with sarcomatous component present in 1% of the neoplasm and rhabdoid component present in 5% of the neoplasm with clear margins (T3bN0M0). He received adjuvant treatment with pembrolizumab, but in January 2023, he complained of progressive lower back pain.

**Fig. 3:**
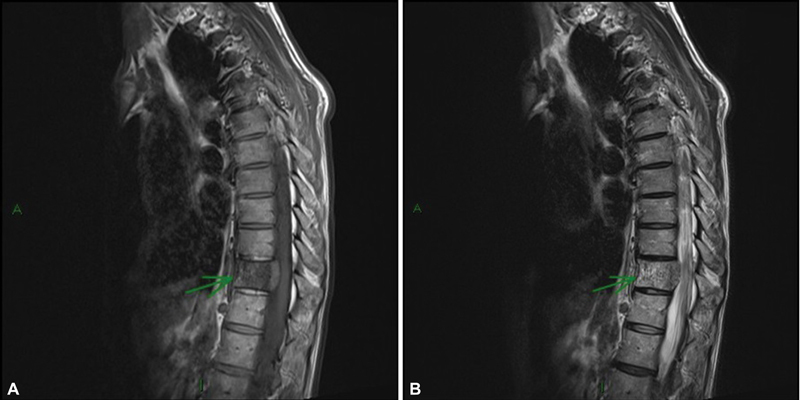
( **A** ) Sagittal magnetic resonance imaging (MRI) section demonstrating iso/hypo-signal in the T1-weighted sequence in the body of the T11 vertebra. ( **B** ) Sagittal MRI section demonstrating heterogeneous signal in the T2-weighted sequence in the body of the T11 vertebra (green arrow).

Initially, laboratory tests and ultrasound of the kidneys and urinary tract were performed, which showed no abnormalities. Subsequently, after refractory pain, an MRI of the spine was requested, which revealed a bone lesion with soft tissue components in the body of T11, with iso/hyposignal in T1 ( Fig. 3A ) and heterogeneous signal in T2 ( Fig. 3B ).

The case was discussed in a tumor board meeting to evaluate the best course of action for the patient at that time. Analyzing the MRI images, hypotheses of atypical/aggressive hemangioma or RC metastasis within a VH (“collision lesion”) were raised, making the precise diagnosis crucial for therapeutic definition.

Based on the characteristics observed in the MRI, and after discussion in the tumor board, hypotheses of atypical/aggressive VH or a “collision lesion,” representing a RC metastasis within a VH, were considered. Furthermore, to avoid unnecessary biopsy, other imaging tests were performed.

**Fig. 4:**
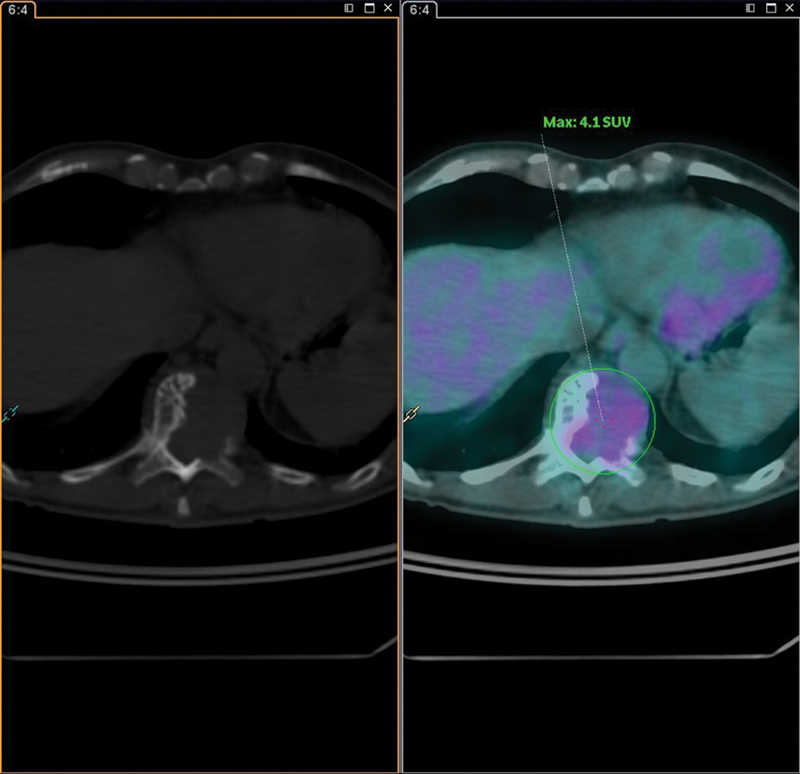
Axial computed tomography slices ( *on the left* ) and 18F-labeled fluorodeoxyglucose positron emission tomography ( *on the right* ) demonstrating an osteolytic lesion with soft tissue component in the body of the T11 vertebra with a maximum standardized uptake value (SUVmax) = 4.1.

**Fig. 5:**
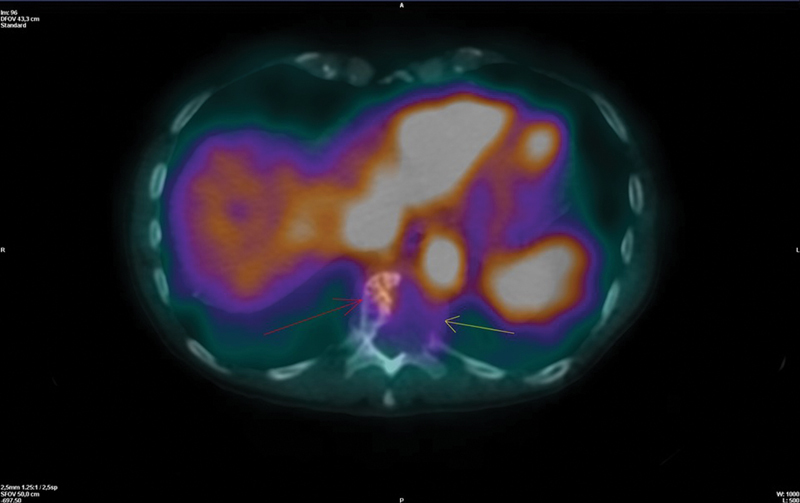
Axial slice of single-photon emission computed tomography/computed tomography fusion from the study of red blood cell scintigraphy showing the uptake of the radiotracer in the remaining bone of the T11 vertebra on the right ( *red arrow* ) and the absence of concentration in the osteolytic area with soft tissue component on the left ( *yellow arrow* ).

**Fig. 6:**
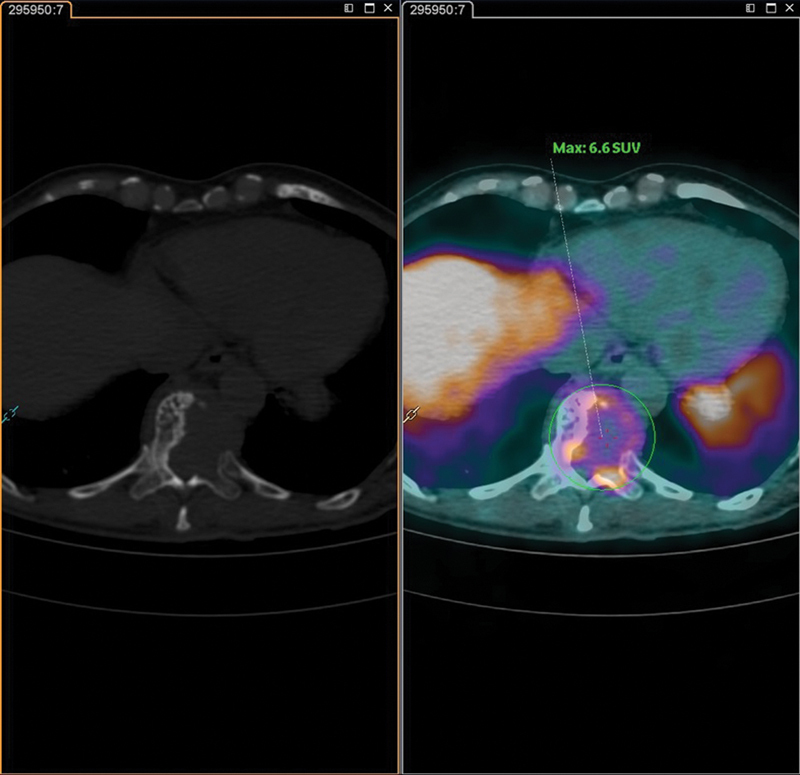
Axial computed tomography slices ( *on the left* ) and prostate-specific membrane antigen prostate-1007 positron emission tomography ( *on the right* ) demonstrating an osteolytic lesion with soft tissue component in the body of the T11 vertebra with maximum standardized uptake value (SUVmax) = 6.6.

The FDG-PET scan revealed an osteolytic lesion in the T11 vertebra with a standardized uptake value (SUV) of 4.1 ( Fig. 4 ). The RBCS showed uptake in the remaining bone of the right T11 vertebra consistent with hemangioma and no concentration in the osteolytic area with soft tissue components on the left ( Fig. 5 ). Additionally, the patient was enrolled in a clinical trial to undergo PSMA-PET study, which showed uptake in the T11 vertebra with an SUV of 6.6 ( Fig. 6 ).

After all the investigations performed, given the results that could not definitively determine the etiology of the vertebral lesion, a biopsy of this area was performed, and the result was ccRC metastasis, characterizing it as a “collision lesion”. Therefore, targeted treatment was initiated.

The proposed treatment for the patient included local radiotherapy for pain control and spinal cord decompression, combined with systemic treatment using nivolumab, cabozantinib, and denosumab. As of our last evaluation, the patient continues to receive multidisciplinary treatment and is stable.

## Discussion

Imaging findings of typical and atypical hemangiomas have been extensively described in the literature. Complications within VHs, such as fractures, and uncommon variants like aggressive hemangiomas, have also been well-documented. However, the presence of another lesion within the hemangioma itself, a collision lesion, is rare, with only three cases previously documented. 9 10 11

VHs are frequent incidental findings on spinal CT and MRI, and when their imaging appearance is typical, they show coarsened vertical trabeculae on radiographic and CT images 6 . On axial unenhanced CT images obtained with a bone algorithm, typical VHs appear as lesions characterized by small punctate areas of high attenuation, representing sparse thickened hyperdense trabeculae surrounded by hypodense stroma, forming the pathognomonic “spotted” or “polka-dot” appearance (polka-dot sign). This appearance simulates the polka-dot pattern on clothing. Typical VHs show a distinctive appearance on MRI due to the presence of serpentine vascular channels and secondary reactive phenomena such as fat overgrowth and bone trabeculae thickening. 12 Fat is usually predominant, so they appear as well-defined and hyperintense lesions on T1- and T2-weighted images. 6 12 Nonetheless, VHs might also display an atypical/aggressive appearance because of their histological features composed of fat, vessels, and interstitial edema. 6 The hyperintense areas represent fat, while the hypointense areas represent flow voids. It has been illustrated that aggressive VHs usually produce a low signal on T1-weighted images and a high signal on T2 and may be confused with the pattern of metastasis, 6 which present as lesions that infiltrate the marrow, replacing medullary fat. 7

The patient presented in this case had lesions in the T5 and T11 vertebrae with tomographic characteristics of hemangioma at the initial staging of RC. However, during treatment, the patient began to experience pain in the thoracic spine, which drew attention since bones are one of the main sites of distant metastases. Therefore, new imaging exams were performed, but the CT revealed an osteolytic lesion in the T11 vertebra, and the MRI supplementation was not entirely clarifying, as the patterns of T1- and T2-weighted images could refer to an atypical hemangioma or RC metastasis.

FDG-PET studies have demonstrated an important role in the diagnosis of many tumors, being the most used radiopharmaceutical. 13 Although the use of FDG-PET is controversial in the primary diagnosis of RC due to urinary excretion of the radiopharmaceutical, its use in restaging is a valuable and well-established tool in the detection of metastatic or recurrent lesions in patients with RC. 14 However, when we performed this study for restaging, we observed only a discreet concentration of the radiopharmaceutical in the osteolytic component of the T11 vertebra, which could mean only a discreet local inflammatory process. This slight uptake may occur in hemangiomas in the PET-FDG study, as previously reported. 15 Additionally, no other suggestive areas of lymph node, bone, or visceral metastasis were identified, which did not allow us to accurately define whether the lesion in T11 was related to RC.

Another diagnostic exam we performed was RBC complemented with single photon emission tomography/computed tomography. In such cases, conventional (non-PET) nuclear medicine techniques such as Tc-99m-labeled RBC may aid in resolving the diagnostic conundrum. 16 Since hemangiomas are blood-filled sinuses, they take up Tc-99m-labeled RBC, differentiating them from other malignant conditions. The images from this study showed concentration of the radiopharmaceutical in the right half of the body of the T11 vertebra, where there was still bone matrix, and absence of concentration in the left half, where an osteolytic component was present. These findings allowed us to infer that the previous hemangioma remained on the right side of the vertebra, and on the left side, there could be a metastatic lesion of RC where the remainder of the hemangioma was previously observed.

Finally, we also performed a PSMA-PET study as part of a clinical research protocol developed at our institution, which prospectively investigates the use of this diagnostic method in the restaging of RC patients. The role of PSMA-PET in evaluating patients with prostate cancer is well known. 17 PSMA is also overexpressed in the neo-vasculature of other tumors, including RC, 18 suggesting that there may be a role for the use of PET/CT with PSMA in this pathology. To date, in the literature, most are case reports and retrospective studies documenting the use of PSMA-PET in the investigation and management of RC patients. 19 Moreover, most of these studies used the Gallium-68 isotope, which undergoes physiological renal excretion, potentially hindering local assessment. Thus, we are developing a prospective study to evaluate the role of PSMA-PET using the fluorine-18 isotope, which undergoes physiological hepatobiliary excretion, with the expectation of better assessing local and metastatic RC lesions. The images from this study showed uptake of ^18^ F-PSMA in the osteolytic component of the T11 vertebra with SUV greater than that observed in the FDG-PET study, which also allowed us to infer the presence of a metastatic lesion from RC.

In summary, our case underscores the intricate diagnostic challenges posed by “collision lesions” in oncology, exemplified by the convergence of ccRC metastasis and a VH. The rarity of such occurrences emphasizes the need for a meticulous and multidisciplinary approach to ensure accurate diagnoses and optimal therapeutic strategies.

The diagnostic journey, navigated through a battery of imaging modalities, including FDG-PET, RBC scintigraphy, and PSMA-PET, illuminated the complexity of distinguishing between atypical hemangiomas and RC metastasis within vertebral lesions. Each diagnostic tool contributed a unique perspective, forming a comprehensive diagnostic mosaic.

Our patient's case highlights the evolving landscape of diagnostic techniques in the realm of RC, particularly the promising role of PSMA-PET using the fluorine-18 isotope. As we navigate this uncharted territory, we acknowledge the ongoing challenges in precisely characterizing lesions and choosing the most appropriate therapeutic interventions.

This case underscores the pivotal role of collaboration among healthcare professionals from diverse specialties in achieving a definitive diagnosis and tailoring effective treatment plans. The nuanced interplay between clinical observations, imaging findings, and advanced diagnostic tools showcases the intricacies of modern oncology practice.

In conclusion, our experience emphasizes the necessity for ongoing vigilance, innovation, and collaboration in the intricate landscape of oncologic diagnostics, especially when confronted with rare and challenging presentations such as “collision lesions.”
